# Relationship of S100 Proteins with Neuroinflammation

**DOI:** 10.3390/biom15081125

**Published:** 2025-08-04

**Authors:** Mario García-Domínguez

**Affiliations:** 1 Program of Immunology and Immunotherapy, Center for Applied Medical Research University of Navarre, 31008 Pamplona, Spain; mgdom@unav.es; 2 Department of Immunology and Immunotherapy, Clínica Universidad de Navarra, 31008 Pamplona, Spain; 3 Centro de Investigación Biomédica en Red de Cáncer (CIBERONC), 28029 Madrid, Spain

**Keywords:** S100 proteins, pro-inflammatory cytokines, CNS, glial cells, EF-hand motif, RAGE

## Abstract

S100 proteins, a family of Ca^2+^-binding proteins, play numerous roles in cellular processes such as proliferation, differentiation, and apoptosis. Recent evidence has highlighted their critical involvement in neuroinflammation, a pathological hallmark of various neurodegenerative disorders including Alzheimer’s disease, multiple sclerosis, and Parkinson’s disease. Among these proteins, S100B and S100A8/A9 are particularly implicated in modulating inflammatory responses in the CNS. Acting as DAMPs, they interact with pattern recognition receptors like RAGE and TLRs, triggering pro-inflammatory signaling cascades and glial activation. While low concentrations of S100 proteins may support neuroprotective functions, increased levels are often associated with exacerbated inflammation and neuronal damage. This review explores the dualistic nature of S100 proteins in neuroinflammatory processes, their molecular interactions, and their potential as biomarkers and therapeutic targets in neurodegenerative disease management.

## 1. Introduction

Within the CNS, neuroinflammation constitutes an intricate response that is now recognized as a crucial driver in the pathogenesis of many neurological disorders [1]. Previously considered a transient protective response to infection, injury, or neurotoxic insult, neuroinflammation is now understood to mediate numerous neuroprotective and neurodegenerative effects, with its impact determined by the specific cellular context, duration of activation, and nature of the triggering factors [2,3].

The dichotomous nature of neuroinflammation is orchestrated by complex interplay between resident glial cells and the molecular signaling pathways within the environment of the CNS [4,5]. Glial cells (mainly microglia) maintain vigilant their local microenvironment during physiological homeostasis [6,7]. In response to pathological stimuli (induced by PAMPs and/or DAMPs), microglia adopt an activated phenotype characterized by the secretion of pro-inflammatory cytokines (e.g., TNF-α, IL-1β, and IL-6), ROS, and NO [8,9,10,11,12]. These responses are orchestrated through intracellular pathways like NF-κB, MAPKs (ERK1/2 and JNK), and the assembly of the NLRP3 inflammasome complex [13,14,15]. PRRs, such as Toll-like receptors (TLRs) and NOD-like receptors (NLRs), serve as proximal sensors that initiate these downstream inflammatory cascades [16,17]. Astrocytes, historically considered passive support cells, are now recognized as active mediators in neuroimmune signaling [18]. Upon stimulation by several microglial-derived mediators including IL-1α, TNF-α, and complement component C1q, astrocytes undergo phenotypic transformation, leading to the loss of neuroprotective functions and the release of neurotoxic molecules that impair neuronal and oligodendrocyte viability [19,20]. Astrocytes also regulate BBB permeability through the secretion of VEGF and MMPs, thereby promoting infiltration of peripheral immune cells like T lymphocytes and monocytes into the CNS parenchyma [21]. Neurons, although traditionally viewed as passive targets of inflammation, play a key role in modulating the CNS immune environment. They express regulatory ligands such as CD200, CX3CL1, and neuregulins, which interact with glial receptors to maintain glial quiescence and support homeostasis [22,23,24]. However, chronic or excessive inflammatory stimulation leads to the suppression of these neuroprotective signals, facilitating a shift toward a sustained pro-inflammatory state [25].

Within the intricate and multifactorial landscape of neuroinflammation in the CNS, the S100 family of proteins has garnered considerable attention as key modulators of glial cell functionality and neuroimmune interactions, comprising a multifaceted area of study that synthesizes principles and methodologies from molecular biology, immunology, and neuroscience [26]. S100 proteins, classified as a family of low-molecular-weight Ca^2+^-binding proteins, are distinguished by the presence of characteristic EF-hand helix-loop-helix Ca^2+^-binding domains and are critically involved in intracellular Ca^2+^ homeostasis and act as versatile intracellular and extracellular signaling mediators [27]. The EF-hand domains confer high-affinity Ca^2+^ binding, inducing some conformational changes that regulate the interaction of S100 proteins with various target molecules, including enzymes, cytoskeletal components, and transcription factors [28,29]. These interactions modulate diverse cellular processes such as proliferation, differentiation, apoptosis, and cytoskeletal dynamics, which are essential for maintaining CNS cellular homeostasis [30,31]. Despite their involvement in CNS function, S100 proteins play crucial roles in maintaining homeostasis by regulating numerous biological processes such as epidermal integrity, hemostasis, angiogenesis, and nutrient uptake [32,33,34,35,36,37,38,39,40,41,42,43,44,45,46,47,48,49,50,51,52,53,54,55,56,57,58,59,60,61,62,63,64,65,66,67,68]. Table 1 presents the members of the S100 protein family, together with their exerted biological functions.

Extracellularly, several S100 proteins are actively secreted or released from activated glial cells through non-classical pathways and act as DAMPs [69]. Upon extracellular release, these S100 proteins engage several PRRs (e.g., RAGE and TLRs), triggering intracellular signaling cascades involving NF-κB and MAPKs [70]. This activation drives the enhanced transcription of some pro-inflammatory cytokines and chemokines, thereby intensifying the neuroinflammatory response [71]. Moreover, intracellular S100 proteins participate in regulating glial cell cytoskeletal reorganization by binding to several cytoskeletal proteins such as tubulin, actin, and intermediate filaments, which affects glial motility and phagocytic capacity during inflammatory responses [72,73,74]. They further modulate intracellular Ca^2+^ dynamics, influencing Ca^2+^-dependent signaling mechanisms, including the calcineurin-NFAT and CaMKII pathways, which are vital for cytokine gene expression and cell survival [75,76]. Collectively, these molecular mechanisms establish S100 proteins as crucial integrators of Ca^2+^-dependent intracellular signaling and extracellular neuroimmune regulation, emphasizing their role in sustaining CNS homeostasis and their contribution to the pathogenesis of neuroinflammatory and neurodegenerative disorders.

Taken together, current evidence positions neuroinflammation as a highly dynamic and multifaceted process that plays a crucial role in both the physiology and pathology of the CNS. The intricate interplay among glial cells, neurons, immune signals, and modulatory proteins such as the S100 family underscores a tightly regulated network that, when disrupted, can lead to highly neurotoxic consequences. A deeper understanding of these mechanisms provides critical insight into the pathogenesis of numerous neurological disorders. Moreover, it opens promising avenues for the development of targeted therapeutic strategies aimed at restoring neuroimmune homeostasis.

This paper will investigate the emerging roles of S100 proteins as pivotal modulators within the neuroinflammatory milieu of the CNS. It will specifically analyze the intracellular and extracellular roles of S100 proteins in modulating glial cell activation, neuroimmune signaling, and cytoskeletal remodeling, with particular emphasis on the integration of these mechanisms within central pro-inflammatory signaling cascades. By integrating current literature and mechanistic insights, this review aims to elucidate how dysregulation of S100-mediated signaling contributes to the onset and persistence of neuroinflammatory states associated with numerous neurological disorders. Ultimately, this work will propose a conceptual framework that identifies S100 proteins as biomarkers and viable therapeutic targets for the modulation of CNS inflammation and the restoration of neuroimmune homeostasis.

## 2. Biology of S100 Proteins

The S100 protein family consists of low-molecular-weight, Ca^2+^-binding proteins containing EF-hand motifs, broadly expressed across numerous cell types. These proteins are involved in several biological processes, including cell proliferation and differentiation, apoptosis, and inflammatory regulation. The elucidation of the molecular characteristics and signaling mechanisms of S100 proteins is crucial for understanding their contributions to both normal physiological functions and disease pathogenesis.

### 2.1. Structural Features of S100 Proteins

**Figure 1:**
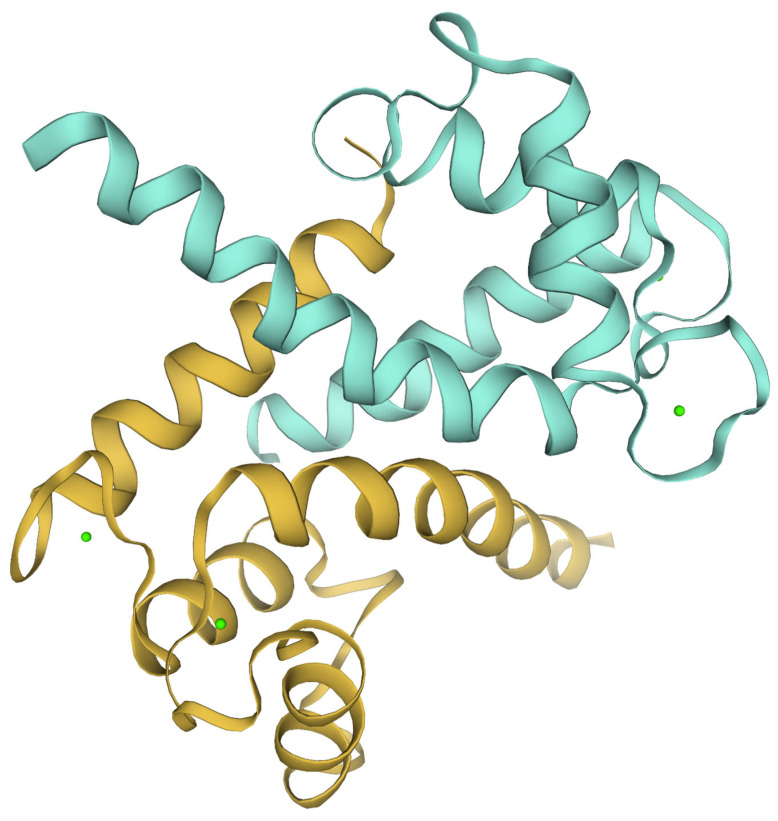
Tridimensional structure of human S100A1 protein. The blue and brown colors delineate the two monomeric subunits constituting the protein, whereas the green spheres depict the bound Ca^2+^ ions. Image generated using Expasy software(3.0).

The S100 protein family (Figure 1) is a subclass of the EF-hand superfamily, a class of Ca^2+^-binding proteins defined by the presence of the highly conserved helix–loop–helix structural motif, termed the EF-hand domain [77]. S100 proteins function as homo- or heterodimers, with each monomer contributing a distinct EF-hand calcium-binding domain. These domains, consisting of approximately 90 to 110 amino acid residues, show extensive sequence conservation and structural homology across the S100 family [78]. A characteristic feature of S100 proteins is the incorporation of two structurally distinct EF-hand Ca^2+^-binding domains within each monomer, with a canonical EF-hand located in the C-terminal region and a non-canonical, or pseudo- EF-hand, placed in the N-terminal region [79].

Each EF-hand motif consists of a 12-residue Ca^2+^-binding loop flanked by two α-helices (E and F helices), which coordinate Ca^2+^ ions through a defined set of side-chain and backbone oxygen ligands [80]. Ca^2+^ coordination typically involves a pentagonal bipyramidal geometry formed by ligands at conserved positions within the loop (e.g., positions 1, 3, 5, 7, 9, and 12) [81]. The two EF-hand motifs are linked by a central flexible hinge region that is essential for the protein’s conformational flexibility [82]. In the absence of Ca^2+^, the S100 proteins adopt a closed conformation with limited target affinity. Upon Ca^2+^ binding, the EF-hand undergoes a significant conformational change driven by reorientation of the flanking helices, enabling interaction with specific target proteins [82]. The Ca^2+^-dependent binding properties of S100 proteins facilitate their role as intracellular sensors and regulators of signaling cascades, mediating interactions with a wide spectrum of intracellular targets (including enzymes, cytoskeletal proteins, transcription factors, and membrane receptors) and consequently modulating downstream cellular processes including proliferation, differentiation, migration, and apoptosis [31,83].

The first S100 protein was characterized by B.W. Moore in 1965, after being extracted from bovine brain tissue [84]. Subsequently, further members of the S100 protein family were isolated from numerous tissues and cell types [85]. The term S100 emerges from their biochemical property of being soluble in a saturated solution of (NH_4_)_2_SO_4_ at neutral pH [84]. Since their initial discovery, the S100 proteins have been the subject of biochemical, molecular, and functional studies. These investigations have demonstrated that S100 proteins are not only evolutionarily conserved but also ubiquitously expressed across a variety of species [86].

In the human genome, the S100 protein family is composed of 20 structurally related but functionally diverse members [87]. The predominant portion of these, specifically 16 isoforms, are encoded by genes that are physically clustered on the long arm of chromosome 1, at cytogenetic band 1q21 [88]. This region comprises a component of the epidermal differentiation complex (EDC) and represents an evolutionarily conserved locus involved in the regulation of epithelial cell differentiation, barrier integrity, and inflammatory processes [89]. Accordingly, the S100 gene cluster is hypothesized to have co-evolved alongside the multifaceted physiological and immunological demands associated with the adaptation of skin in terrestrial vertebrates [90,91]. The remaining S100 family members are encoded by those genes positioned on separate chromosomal loci, indicating that gene duplication and diversification events have played a significant role in the evolutionary history of this protein family [92].

A hallmark characteristic of the S100 protein family is their selective tissue- and cell-type-specific expression patterns [93]. This regulation is orchestrated through numerous mechanisms, encompassing transcriptional control by some transcription factors and epigenetic modifications, alongside post-transcriptional processes including mRNA stability modulation, alternative splicing, and microRNA-mediated repression [94]. These regulatory mechanisms ensure context-dependent expression of S100 proteins, enabling them to perform some functions in cellular physiology and intercellular communication [94]. Although S100 protein expression is incredibly regulated under physiological conditions, its dysregulation frequently occurs as a hallmark in various pathological contexts, especially during oncogenesis [95,96].

### 2.2. S100 Protein Receptors

The interaction between S100 proteins and their principal receptor, RAGE (Receptor for Advanced Glycation End-products), represents a critical molecular axis in both physiological and pathological signaling, especially in the context of inflammation, cancer, and neurodegeneration [97,98,99]. This interaction is governed by complex Ca^2+^-dependent conformational rearrangements, oligomerization dynamics, and receptor-ligand recognition mechanisms, ultimately leading to the activation of some intracellular signaling cascades with downstream consequences [100]. Binding of Ca^2+^ to S100 proteins evokes a conformational rearrangement that unmasks hydrophobic regions essential for interaction with the RAGE receptor [101]. This Ca^2+^-dependent exposure of functional interfaces is further fine-tuned by Zn^2+^ ions, which, in S100 family members such as S100A8 and S100A9, enhance structural stability and augment receptor-binding affinity, likely by promoting oligomerization and increasing ligand valency [102].

Following their secretion into the extracellular milieu, S100 proteins such as S100A4, S100B, and the S100A8/A9 heterodimer interact with the extracellular V-type IgG domain of RAGE, initiating ligand–receptor recognition at the cell surface [103]. This interaction is dependent on the oligomeric configuration of the S100 ligands; notably, S100A8/A9 forms higher-order oligomers that enable multivalent engagement with RAGE, promoting receptor oligomerization and potentiation of downstream signaling [103].

**Figure 2:**
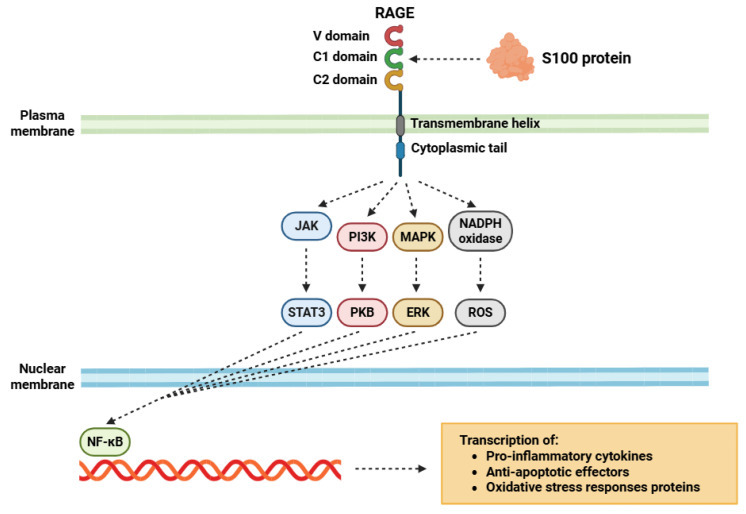
Diagram that illustrates the activation process of RAGE receptors following the binding of S100 proteins. Abbreviations: RAGE (receptor for advanced glycation end products), JAK (Janus kinase), STAT3 (signal transducer and activator of transcription 3), PI3K (phosphoinositide 3-kinase), PKB (protein kinase B), MAPK (mitogen-activated protein kinase), ERK (extracellular signal-regulated kinase), NADPH (nicotinamide adenine dinucleotide phosphate), ROS (reactive oxygen species), and NF-κB (nuclear factor kappa-light-chain-enhancer of activated B cells).

At the intracellular level, RAGE (Figure 2) lacks intrinsic enzymatic activity, like tyrosine kinase or serine/threonine kinase function [104]. As a result, RAGE recruits several adaptor proteins to initiate and propagate intracellular signaling cascades [105]. A fundamental adaptor in this context is diaphanous-related formin-1 (DIAPH1), which associates with the cytoplasmic domain of RAGE, mainly mediated by interactions with a conserved motif situated in the intracellular domain of the receptor [106]. This interaction is usually induced or stabilized following ligand-mediated RAGE oligomerization at the plasma membrane. The RAGE-DIAPH1 complex serves as a scaffold for the assembly of multiprotein signalosomes, which integrate and transduce extracellular cues into defined intracellular signaling events [107]. One of the well-characterized downstream signaling pathways activated via this mechanism is the nuclear factor kappa-light-chain-enhancer of activated B cells (NF-κB) cascade [108]. Upon RAGE engagement by S100 ligands, the receptor–adaptor complex facilitates the recruitment and activation of the IκB kinase (IKK) complex, composed of IKKα, IKKβ, and the regulatory subunit IKKγ (designed as NEMO) [109].

Activation of IKKβ leads to the phosphorylation of IκBα, the inhibitory protein that sequesters NF-κB dimers (usually p50/p65) in the cytoplasm under basal conditions [110]. Phosphorylated IκBα is recognized by the SCF(βTrCP) E3 ubiquitin ligase complex, leading to its polyubiquitination and subsequent degradation by the 26S proteasome complex [111]. The proteasomal degradation of IκBα reveals the nuclear localization sequences of the NF-κB subunits, facilitating their subsequent nuclear translocation into the nucleus through importin-mediated transport [112]. Once translocated into the nucleus, NF-κB interacts with κB response elements within the promoter regions of its target genes, promoting the transcription of numerous genes involved in immune regulation [113,114]. Among the transcriptional targets are pro-inflammatory cytokines (such as TNF-α, IL-1β, and IL-6), adhesion molecules (such as ICAM-1 and VCAM-1), anti-apoptotic effectors (such as Bcl-xL), and those enzymes implicated in oxidative stress responses, such as iNOS and COX-2 [115,116,117,118].

Beyond NF-κB, the RAGE-DIAPH1 axis also interfaces with small GTPase signaling (e.g., RhoA and Rac1), thereby affecting cell motility, vascular permeability, and the reorganization of the cytoskeleton [119]. Moreover, RAGE can engage other adaptor proteins (or co-receptors) to activate parallel signaling cascades including MAPK pathways (such as ERK1/2, p38, and JNK) which modulate gene expression, cellular differentiation, and stress responses [120]; PI3K/PKB signaling, contributing to cell survival, proliferation, and metabolic adaptation [121]; JAK/STAT3 activation, indirectly mediated via IL-6 signaling downstream of NF-κB, sustains a feed-forward inflammatory loop that promotes the pro-inflammatory response [122].

The specificity, affinity, and functional outcome of the interaction between S100 proteins and RAGE are not determined solely by the presence of divalent cations such as Ca^2+^ and Zn^2+^, but are also regulated by a spectrum of post-translational modifications (PTMs) that affect ligand and receptor [123]. These PTMs act as regulatory molecular switches that dynamically integrate environmental stimuli (such as oxidative stress, metabolic alterations, and inflammatory signals) into finely tuned biochemical interactions with distinct cellular consequences [124]. On the ligand side, several members of the S100 protein family (particularly S100A8 and S100A9) harbor redox-sensitive cysteine residues, which are susceptible to oxidative modifications under conditions of increased ROS, a common feature of inflamed or metabolically dysregulated tissues [125]. These cysteine residues can undergo S-glutathionylation, S-nitrosylation, or form inter- and intramolecular disulfide bonds, which in turn influence the protein’s oligomeric configuration, structural stability, and receptor interaction affinity [126,127]. In contrast, excessive oxidation might disrupt receptor interaction by inducing aberrant conformations or promoting protein aggregation [128].

In addition to redox-based regulation, phosphorylation of S100 proteins (principally at serine and threonine residues located in flexible loops or C-terminal regions) can impact their conformational dynamics, subcellular localization, and secretion [129]. Kinases (such as PKC and CK2) are critically involved in mediating the phosphorylation of S100 proteins upon particular stimulatory signals [130,131]. These phosphorylation events can promote or inhibit RAGE binding by altering the electrostatic potential and steric accessibility of receptor-interacting surfaces [100]. On the receptor side, RAGE is subject to extensive N-linked glycosylation at multiple asparagine residues within its extracellular domains. This glycosylation is critical for maintaining proper receptor folding, surface expression, and ligand recognition [132]. Furthermore, RAGE glycoforms may exhibit differential affinities for specific S100 ligands, contributing to cell type- and context-specific signaling outputs [133].

### 2.3. Biological Functions of S100 Proteins

S100 proteins perform a broad spectrum of intracellular functions (Table 1), primarily acting as Ca^2+^ sensors or Ca^2+^-dependent regulatory molecules [86]. Upon Ca^2+^ binding, S100 proteins undergo conformational rearrangements that expose hydrophobic surfaces, facilitating interactions that modulate the activity, localization, or stability of key intracellular components, thereby influencing several physiological processes, including the regulation of the cell cycle and cellular proliferation [101,134]. In the context of differentiation, some S100 proteins contribute to the regulation of lineage-specific gene expression and differentiation processes, particularly within neural and myogenic lineages, indicating a functional involvement in developmental programming and the modulation of tissue-specific plasticity [32,135]. S100 proteins also play a key role in maintaining cytoskeletal dynamics and promoting cell motility. Through direct binding to actin-associated proteins, including non-muscle myosin IIA, tropomyosin, and tubulin, several S100 proteins contribute to cellular architecture remodeling, adhesion dynamics, and directional migration [37,136]. In the context of cell fate regulation, these proteins show several pro-apoptotic functions under both physiological conditions and oxidative stress [137,138]. At the transcriptional level, S100 proteins modulate gene expression either through direct interaction with transcription factors like NF-κB, HIF-1α, and p53, or indirectly via Ca^2+^-dependent signaling pathways, thereby orchestrating cellular responses to differentiation signals, inflammatory cues, and oxidative stress [75,108,139,140].

Despite lacking classical secretion signals, several S100 proteins are secreted or released into the extracellular environment via non-classical mechanisms in response to cellular activation, injury, or necrosis. S100 proteins serve as strong pro-inflammatory mediators as a result of their categorization among DAMPs [83]. Binding of S100 proteins to these receptors initiate downstream signaling cascades, including the NF-κB and MAPK pathways, culminating in the production and secretion of several pro-inflammatory cytokines, including TNF-α, IL-1β, and IL-6 [29,70]. These pro-inflammatory cytokines act synergistically to activate some immune cell populations (e.g., neutrophils, monocytes, and macrophages) into the site of inflammation, thereby perpetuating tissue damage [141]. In addition to TNF-α, IL-1β, and IL-6, other cytokines such as IL-8, IL-17, and GM-CSF are also upregulated in response to extracellular S100 protein signaling [142,143,144]. IL-8 plays a critical role in neutrophil chemotaxis and activation [145], while IL-17 further enhances neutrophil recruitment and promotes sustained inflammation [146]. GM-CSF contributes to the differentiation of myeloid lineage cells, thus supporting a prolonged inflammatory environment [147]. Collectively, these cytokines orchestrate a complex and highly regulated inflammatory cascade that is central to the pathophysiology of various chronic inflammatory and autoimmune conditions.

Furthermore, extracellular S100 proteins are implicated in the modulation of immune cell migration and chemotactic responses [148]. Some S100 proteins function as a chemoattractant that promotes the recruitment of neutrophils, monocytes, and macrophages to sites of tissue injury or infection, playing a key role in innate immune defense [149]. In the context of tissue remodeling and repair, S100 proteins regulate extracellular matrix (ECM) turnover by modulating the expression and enzymatic activity of matrix metalloproteinases (MMPs), facilitating several processes such as wound healing, fibrosis, and angiogenesis [55,150,151]. Reduced expression or functional impairment of specific S100 proteins has been observed in conditions like Alzheimer’s disease (AD) [152], Parkinson’s disease (PD) [153], inflammatory diseases [154], and several cancers [155], indicating their crucial involvement in pathophysiological mechanisms.

## 3. Role of S100 Proteins in Neuroinflammation

### 3.1. Contribution of S100 Proteins to Neuroinflammatory Mechanisms

In the CNS, S100 proteins are increasingly recognized for their crucial roles not only in the regulation of homeostatic processes but also in the amplification and perpetuation of neuroinflammatory responses under physiopathological conditions [30,31]. Within the S100 family, S100A1, S100A6, S100A8/9, S100A12, and S100B show prominent expression and functional relevance within the CNS [156,157,158,159,160]. These proteins are differentially localized across several CNS-resident cell types, like astroglia, microglia, oligodendroglia, and neurons, with expression levels and localization patterns regulated in a cell-type, region-specific, and temporally dynamic manner [156,157,158,159,160]. Astrocytes are the principal source of S100B, although they also express S100A1 and S100A6 under basal and activated states [161,162,163]. Microglia express inducible S100 proteins like S100A8/9, and S100A12, mainly under pro-inflammatory stimuli [164,165]. Oligodendrocytes, less studied in the context of S100 protein biology, express S100A6 and possibly S100B during development and in demyelinating conditions [166]. Neurons produce smaller levels of S100 proteins under normal conditions, although upregulation has been reported in response to injury or excitotoxic stress [167].

However, the functional profile of S100 proteins drastically changes under pathological conditions characterized by CNS injury, infection, ischemia, or neurodegeneration. In these states, S100 proteins are usually overexpressed and released into the extracellular space due to enhanced cellular stress, damage, or death [168]. Upon release into the extracellular environment, S100 proteins act as DAMPs, initiating and sustaining neuroinflammatory responses [83]. The downstream signaling cascades triggered by RAGE activation led to the transcriptional upregulation of many pro-inflammatory cytokines (such as TNF-α, IL-1β, and IL-6), chemokines (e.g., CCL2 and CXCL10), and adhesion molecules (like ICAM-1 and VCAM-1), which collectively orchestrate the recruitment and activation of immune cells, including resident microglia and infiltrating leukocytes [115,116,117,118].

The cell-specific expression profiles of S100 proteins within the CNS are closely associated with their distinct functional roles. For instance, S100B, which is highly abundant in mature astrocytes, is involved in cytoskeletal reorganization, regulation of intracellular calcium signaling, modulation of energy metabolism, and maintenance of the extracellular matrix [169]. It acts as a neurotrophic factor at low concentrations, promoting neuronal survival, synaptic plasticity, and neurite outgrowth [74]. In contrast, at higher extracellular concentrations, S100B assumes a pro-inflammatory and neurotoxic role, thereby contributing to reactive gliosis, microglial activation, and disruption of the BBB [169]. Moreover, chronic exposure to high extracellular S100B has been associated with impaired synaptic plasticity, increased oxidative stress, and neuronal apoptosis [74]. S100A1, primarily expressed in neurons and astrocytes, contributes to intracellular Ca^2+^ homeostasis, mitochondrial integrity, and modulation of neurotransmitter release. It is believed to be involved in synaptic function and long-term potentiation (LTP), mechanisms underlying learning and memory [170]. S100A6 is localized in astrocytes and neurons and might be involved in protein degradation pathways, cellular proliferation, and stress responses, although its roles in the CNS remain under investigation [171]. The pro-inflammatory members of the family (S100A8, S100A9, and S100A12) are recognized as “alarmins” or DAMPs that are rapidly upregulated in microglia and astrocytes upon exposure to pathological stimuli, incl4uding trauma, ischemia, or pathogenic infection [172].

In response to acute CNS injuries, including traumatic brain injury (TBI) or ischemic stroke, the expression of S100 proteins (most notably S100B, S100A1, and S100A8/A9) is markedly upregulated, reaching maximal levels within hours to several days after the insult [173,174]. This acute-phase upregulation is predominantly driven by astrocytes and microglia, which release S100 proteins into the extracellular space in response to cytokine signaling and oxidative stress [161,162,163,164]. In chronic neurodegenerative diseases like AD, PD, multiple sclerosis (MS), and amyotrophic lateral sclerosis (ALS), S100 proteins exhibit persistently elevated expression and accumulate in brain regions marked by neuronal degeneration, gliosis, and chronic inflammation [152,153,175,176,177]. This prolonged upregulation fosters a self-perpetuating cycle of chronic, low-grade neuroinflammation, synaptic impairment, and progressive neuronal loss [152,153,175,176,177].

A critical aspect of S100 protein function in the CNS lies in their spatial and temporal expression patterns [178]. Transient and localized expression following acute injury may support protective responses such as debris clearance and tissue repair [179]. However, sustained and widespread expression in chronic conditions usually leads to maladaptive consequences, like synaptic loss, neuronal death, and persistent neuroinflammation [180].

### 3.2. Role of S100 Proteins in Alzheimer’s Disease

In AD, the Ca^2+^-binding protein S100B is markedly overexpressed by reactive astrocytes, particularly in the vicinity of β-amyloid (Aβ) plaques [181]. Increased extracellular concentrations of S100B, often reaching micromolar levels, initiate a pathological cascade via binding to the RAGE receptor on neighboring neurons and glial cells [182]. This engagement activates the Ras-MAPK signaling pathway, principally ERK1/2, which leads to enhanced transcription and translation of β-site APP-cleaving enzyme 1 (BACE1), a key enzyme in the amyloidogenic cleavage of amyloid precursor protein (APP) [183]. Moreover, S100B promotes stabilization and activity of BACE1 through post-translational modifications involving Ca^2+^/calmodulin-dependent kinases [181]. As a result, APP processing is diverted from the non-amyloidogenic α-secretase pathway toward β-secretase-mediated cleavage, facilitating increased production and extracellular deposition of neurotoxic Aβ peptides [184]. Moreover, S100B has been shown to induce hyperphosphorylation of τ through activation of ERK1/2 and p38 MAPK pathways, contributing to the detachment of τ from microtubules and the formation of neurofibrillary tangles [185]. These τ species exacerbate cytoskeletal instability and propagate tau pathology [186]. Furthermore, other members of the S100 family, such as S100A8/9, are upregulated in AD and can form heterodimers that directly interact with Aβ peptides [187,188,189]. These heterodimers improve Aβ aggregation and promote the maturation of amyloid plaques [187,188,189].

### 3.3. Role of S100 Proteins in Parkinson’s Disease

In PD, S100B levels are significantly increased in the substantia nigra pars compacta (SNpc), where they correlate spatially and temporally with the progressive loss of dopaminergic neurons [190,191]. The principal source of increased S100B is reactive astrocytes, which release the protein into the extracellular space in response to mitochondrial dysfunction, oxidative stress, or pro-inflammatory stimuli [192]. Extracellular S100B binds to RAGE on microglia and neurons, triggering NF-κB-mediated transcriptional activation of inducible nitric oxide synthase (iNOS), cyclooxygenase-2 (COX-2), and pro-inflammatory cytokines such as TNF-α and IL-1β [153]. This cascade results in increased production of NO and ROS, which subsequently induce mitochondrial dysfunction (mainly through impairment of complex I) disrupt ATP synthesis, and promote oxidative modifications that facilitate α-synuclein aggregation [193,194]. In dopaminergic neurons, the oxidative environment promotes the formation of peroxynitrite, leading to nitrosative stress, collapse of the mitochondrial membrane potential, and subsequent release of cytochrome c, which robustly activates apoptotic signaling pathways [195,196]. Notably, S100B gene variants associated with higher expression have been linked to earlier PD onset, and pharmacological inhibition of S100B–RAGE signaling (e.g., via pentamidine) has shown neuroprotective effects in experimental models, suggesting that this pathway contributes causally to disease progression [197].

### 3.4. Role of S100 Proteins in Multiple Sclerosis

**Figure 3:**
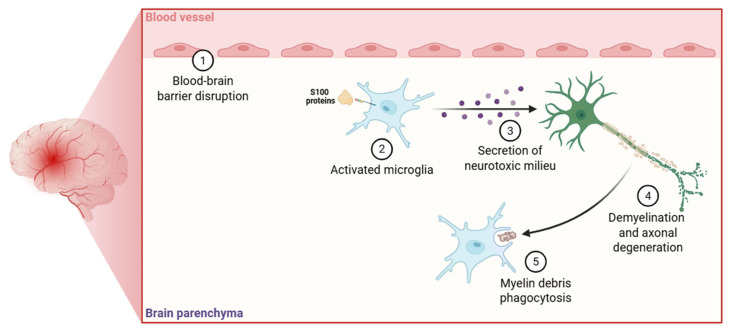
Schematic illustration depicting the role of S100 proteins in microglial activation. Following stimulation by S100 proteins, microglia mediate the breakdown of myelin sheaths, ultimately contributing to the pathogenesis of MS.

In MS (Figure 3), S100A8/9 proteins are markedly upregulated in active demyelinating lesions and are detectable at increased levels in the blood serum of patients during relapses [198]. These proteins, principlly released by activated microglia and infiltrating monocytes, function as DAMPs that enhance neuroinflammatory signaling cascades [69]. Upon extracellular release, S100A8/A9 heterodimers interact with TLR4 and RAGE on resident glial cells, activating the MyD88-dependent signaling cascade, which culminates in NF-κB and p38 MAPK activation [29,158]. This process drives the transcription of pro-inflammatory chemokines, including CCL2 and CXCL10, which mediate the recruitment of peripheral immune cells (principally Th1 and Th17 cells) to demyelinated regions, thereby exacerbating myelin degradation and promoting axonal injury [199,200]. Conversely, S100A8/A9 promote apoptosis in oligodendrocyte precursor cells (OPCs) through the induction of pro-inflammatory cytokine production by microglia [163].

### 3.5. Role of S100 Proteins in Another Neuroinflammatory Diseases

In the context of ALS, some proteins of the S100 family have emerged as critical molecular mediators in the disease’s multifaceted pathophysiology [201]. Some studies have documented the accumulation of S100B and S100A6 in both astrocytes and motor neurons within the spinal cord of ALS patients and animal models, implicating it in neurodegenerative processes that characterize this fatal motor neuron disease [162,177,202,203,204,205]. Notably, the upregulation of S100B has been associated with a maladaptive cellular stress response, particularly in the context of oxidative stress, which is a characteristic hallmark of ALS [206]. On the other hand, recent findings suggest that S100A8/A9 may also interact with mutant superoxide dismutase 1 (SOD1), an enzyme contributing to ALS-related neurodegenerative processes [205]. Finally, S100B has been implicated in the modulation of the BBB integrity, which is often compromised in ALS [207,208].

Finally, S100B protein play a pivotal role in the molecular response to TBI [209]. After injury, astrocytes release S100B into the extracellular space and cerebrospinal fluid (CSF), a process that reflects active secretion in response to metabolic stress and passive leakage from damaged or necrotic brain tissue [210,211]. Furthermore, other members of the S100 family, including S100A4, S100A6, and S100A8 have been detected in astrocytes and neurons and may contribute to post-injury disturbances in intracellular Ca^2+^ signaling [212,213,214]. These proteins are also implicated in secondary injury mechanisms, potentially modulating oxidative stress, neuroinflammatory pathways, and glial cell activation, thereby complementing the pathological and diagnostic relevance of S100B in the context of TBI [212,213,214].

In summary, the roles of S100 proteins in the CNS are highly context-dependent, governed by their spatial distribution, cellular origin, concentration, and extracellular receptor engagement. Under physiological conditions, S100 proteins support critical processes including neurogenesis, synaptic function, and Ca^2+^ homeostasis. Under pathological circumstances, however, S100 proteins act as potent pro-inflammatory mediators, amplifying glial activation, oxidative stress, and neuronal injury. This duality positions S100 proteins as key mediators of CNS function and homeostasis, and as potential drivers of neuropathology when their regulation is disrupted. As a result, S100 proteins function as key endogenous amplifiers of neuroimmune activity and as emerging biomarkers of CNS pathology, offering significant potential as targets for therapeutic intervention in numerous neuroinflammatory and neurodegenerative diseases.

## 4. Conclusions

S100 proteins play multifaceted and dynamic roles in the regulation of neuroinflammatory processes, demonstrating their complex expression dynamics and diverse functional roles within the CNS. These small Ca^2+^-binding proteins, characterized by the EF-hand motif, act in both intracellular and extracellular contexts to modulate a wide range of physiological and pathological mechanisms. Notably, members like S100B, S100A8/A9, and S100A12 have emerged as pivotal molecular mediators at the interface between neuronal function and immune system activation [29]. Intracellularly, S100 proteins participate in Ca^2+^ homeostasis and cytoskeletal organization, among others [72,73,74], whereas in the extracellular environment, they can act as DAMPs, activating several innate immune responses [69,70].

A key feature of extracellular S100 proteins is their interaction with PRRs, mainly the RAGE and TLR4 receptors. These interactions initiate and sustain pro-inflammatory signaling cascades, leading to the activation of transcription factors such as NF-κB and subsequent expression of cytokines, chemokines, and adhesion molecules [69]. This signaling contributes to the recruitment and activation of microglia and astrocytes, promotes disruption of the BBB, and enhances leukocyte infiltration, ultimately aggravating neuroinflammatory states [169]. The capacity of S100 proteins to sustain inflammatory signaling positions them as central regulators of both physiological immune surveillance and pathological inflammation within the CNS.

Importantly, dysregulated expression and secretion of S100 proteins have been consistently associated with the pathogenesis and progression of several major neurodegenerative disorders, such as AD, PD, and MS [152,153,175,176,177]. Despite significant progress in understanding the role of S100 proteins in CNS inflammation, substantial gaps remain in our knowledge. The molecular mechanisms by which specific S100 isoforms regulate glial cell phenotypes, BBB permeability, oxidative stress, and synaptic plasticity are not fully elucidated. Furthermore, the temporal dynamics of S100 protein expression during the progression from acute injury to chronic neuroinflammation are poorly characterized, which limits the ability to exploit these proteins for therapeutic timing and stratification.

Future studies should aim to elucidate the roles of S100 proteins with greater resolution by integrating single-cell transcriptomics, proteomics, and in vivo imaging of inflammatory processes. Particular emphasis should be placed on isoform-specific functions, post-translational modifications, and the impact of both cellular and systemic contexts on S100 protein expression and activity. In parallel, the development of selective inhibitors, decoy receptors, and neutralizing antibodies targeting S100-RAGE or S100-TLR4 interactions holds great promise for therapeutic modulation of neuroinflammatory responses. Finally, the potential of S100 proteins as diagnostic biomarkers warrants further validation in longitudinal clinical studies, especially in relation to disease onset, progression, and therapeutic response.

In conclusion, the S100 protein family represents a key underexplored component of neuroinflammatory regulation. Their biological actions and central position in CNS immune signaling make them attractive targets for both mechanistic studies and therapeutic intervention. A more comprehensive understanding of their roles across diverse neuroinflammatory conditions may facilitate the development of novel strategies to attenuate the detrimental effects of chronic CNS inflammation and to enhance neuroprotection and repair.

## Figures and Tables

**Table 1:** List of S100 protein family members. Abbreviations: Ca^2+^ (calcium ion), p53 (tumor protein p53), FGF1 (fibroblast growth factor 1), IL-1α (interleukin 1 alpha), and CNS (central nervous system).

S100 Protein Member	Chromosomal Location	Functions	References
S100A1	1q21.3	Modulates contractility	[32]
		Regulates Ca^2+^ handling in heart and skeletal muscle	[33]
S100A2	1q21.3	Involved in p53-mediated cell cycle arrest and tumor suppression	[34]
		Negatively impacts tissue repair	[35]
S100A3	1q21.3	Involved in hair shaft formation	[36]
S100A4	1q21.3	Promotes cell motility, invasion, and metastasis	[37,38]
S100A5	1q21.3	Modulation of neuronal activity	[39]
S100A6	1q21.3	Regulates cytoskeletal dynamics and proliferation	[40]
(Calcyclin)			
S100A7	1q21.3	Antimicrobial peptide	[41]
(Psoriasin)		Its overexpression is linked to psoriasis and breast cancer progression	[42,43]
S100A8/9	1q21.3	Pro-inflammatory and antimicrobial roles	[44,45]
(Calprotectin)			
S100A10	1q21.3	Regulates membrane trafficking and plasminogen activation	[46,47]
S100A11	1q21.3	Involved in cell proliferation, motility, and Ca^2+^ signal transduction	[48,49,50]
S100A12	1q21.3	Associated with inflammatory diseases	[51]
(Calgranulin C)		Inducer of neurite growth	[52]
S100A13	1q21.3	Mediates non-classical secretion of FGF1 and IL-1α	[53,54]
		Involved in angiogenesis and cellular stress responses	[55,56]
S100A14	1q21.3	Influences cell proliferation and apoptosis: dual role in cancer	[57,58]
S100A15	1q21.3	Involved in skin immune response	[59]
S100A16	1q21.3	Implicated in adipocyte differentiation and tumor progression	[60,61]
S100B	21q22.3	Regulates cell proliferation and apoptosis	[62,63]
		Marker of CNS injury	[64]
S100G	Xp22.2	Involved in intestinal Ca^2+^ absorption	[65]
(Calbindin-D_9k_)			
S100P	4p16.1	Promotes tumor progresion and metastasis	[66,67]
(Placental S100)			
S100Z	5q13.3	Interacts with S100P	[68]

## Data Availability

Not applicable. No new data were generated.

## Abbreviations

(NH_4_)_2_SO_4_	Ammonium sulfate
AD	Alzheimer’s disease
APP	Amyloid precursor protein
Aβ	Beta-amyloid
BACE1	β-site APP-cleaving enzyme 1
BBB	Blood-brain barrier
Bcl-xL	B-cell lymphoma-extra large
C1q	Complement component 1q
Ca^2+^	Calcium ion
CaMKII	Calcium/calmodulin-dependent protein kinase II
CCL2	C-C motif chemokine ligand 2
CD200	Cluster of differentiation 200
CK2	Casein kinase 2
CNS	Central nervous system
COX-2	Cyclooxygenase 2
CSF	Cerebrospinal fluid
CX3CL1	Chemokine (C-X3-C motif) ligand 1
CXCL10	C-X-C motif chemokine ligand 10
DAMP	Damage-associated molecular patterns
DIAPH1	Diaphanous-related formin-1
ECM	Extracellular matrix
EDC	Epidermal differentiation complex
EF-hand	Helix–loop–helix structural motif for Ca^2+^ binding
ERK	Extracellular signal-regulated kinase
ERK1/2	Extracellular signal-regulated kinases 1 and 2
FGF1	Fibroblast growth factor 1
GM-CSF	Granulocyte-macrophage colony-stimulating factor
HIF-1α	Hypoxia-Inducible Factor 1-alpha
ICAM-1	Intercellular adhesion molecule 1
IgG	Immunoglobulin G
IKK	IκB kinase
IL-1α	Interleukin 1 alpha
IL-1β	Interleukin 1 beta
IL-17	Interleukin 17
IL-6	Interleukin 6
IL-8	Interleukin 8
iNOS	Inducible nitric oxide synthase
JAK	Janus kinase
JNK	c-Jun N-terminal kinase
LTP	Long-term potentiation
MAPK	Mitogen-activated protein kinase
MMP	Matrix metalloproteinase
MS	Multiple sclerosis
MyD88	Myeloid differentiation primary response 88
NADPH	Nicotinamide adenine dinucleotide phosphate
NEMO	NF-κB essential modulator (regulatory subunit IKKγ)
NFAT	Nuclear factor of activated T-cell
NF-κB	Nuclear factor kappa-light-chain-enhancer of activated B cells
NLR	NOD-like receptors
NLRP3	NOD-, LRR- and pyrin domain-containing protein 3
NO	Nitric oxide
OPC	Oligodendrocyte precursor cell
p53	Tumor protein p53
PAMPs	Pathogen-associated molecular patterns
PD	Parkinson’s disease
PI3K	Phosphoinositide 3-kinase
PKB	Protein kinase B
PKC	Protein kinase C
PRR	Pattern recognition receptor
PTM	Post-translational modification
RAGE	Receptor for advanced glycation end-products
Ras-MAPK	Ras-mitogen-activated protein kinase
ROS	Reactive oxygen species
S100	S100 protein family
SCF(βTrCP)	Skp, cullin, F-box containing complex (β-TrCP subunit)
SNpc	Substantia nigra pars compacta
sRAGE	Soluble receptor for advanced glycation end products
STAT3	Signal transducer and activator of transcription 3
TBI	Traumatic brain injury
Th1	T helper type 1 cells
Th17	T helper type 17 cells
TLR	Toll-like receptor
TLR4	Toll-like receptor 4
TNF-α	Tumor necrosis factor alpha
VCAM-1	Vascular cell adhesion molecule 1
VEGF	Vascular endothelial growth factor
Zn^2+^	Zinc ion
τ	Tau protein

## References

[1] Kölliker-Frers R., Udovin L., Otero-Losada M., Kobiec T., Herrera M.I., Palacios J., Razzitte G., Capani F. (2021). Neuroinflammation: An Integrating Overview of Reactive-Neuroimmune Cell Interactions in Health and Disease. Mediat. Inflamm..

[2] Ceulemans A.G., Zgavc T., Kooijman R., Hachimi-Idrissi S., Sarre S., Michotte Y. (2010). The dual role of the neuroinflammatory response after ischemic stroke: Modulatory effects of hypothermia. J. Neuroinflamm..

[3] Kim M.E., Lee J.S. (2024). Mechanisms and Emerging Regulators of Neuroinflammation: Exploring New Therapeutic Strategies for Neurological Disorders. Curr. Issues Mol. Biol..

[4] Shabab T., Khanabdali R., Moghadamtousi S.Z., Kadir H.A., Mohan G. (2017). Neuroinflammation pathways: A general review. Int. J. Neurosci..

[5] Di Vito A., Donato G., Tomassoni D. (2017). Molecular and Cellular Mechanisms of Neuroinflammation. Biomed. Res. Int..

[6] Afridi R., Bhusal A., Tsuda M., Ryu H., Suk K. (2023). Function of Glial Cells in Neuroinflammatory and Neuroimmunological Responses II. Cells.

[7] Muzio L., Viotti A., Martino G. (2021). Microglia in Neuroinflammation and Neurodegeneration: From Understanding to Therapy. Front. Neurosci..

[8] Figuera-Losada M., Rojas C., Slusher B.S. (2014). Inhibition of microglia activation as a phenotypic assay in early drug discovery. J. Biomol. Screen.

[9] Gülke E., Gelderblom M., Magnus T. (2018). Danger signals in stroke and their role on microglia activation after ischemia. Ther. Adv. Neurol. Disord..

[10] Smith J.A., Das A., Ray S.K., Banik N.L. (2012). Role of pro-inflammatory cytokines released from microglia in neurodegenerative diseases. Brain Res. Bull..

[11] Simpson D.S.A., Oliver P.L. (2020). ROS Generation in Microglia: Understanding Oxidative Stress and Inflammation in Neurodegenerative Disease. Antioxidants.

[12] Liy P.M., Puzi N.N.A., Jose S., Vidyadaran S. (2021). Nitric oxide modulation in neuroinflammation and the role of mesenchymal stem cells. Exp. Biol. Med..

[13] Zhou Y., Cui C., Ma X., Luo W., Zheng S.G., Qiu W. (2020). Nuclear Factor κB (NF-κB)-Mediated Inflammation in Multiple Sclerosis. Front. Immunol..

[14] Ji R.R., Suter M.R. (2007). p38 MAPK, microglial signaling, and neuropathic pain. Mol. Pain.

[15] Chen Y., Ye X., Escames G., Lei W., Zhang X., Li M., Jing T., Yao Y., Qiu Z., Wang Z. (2023). The NLRP3 inflammasome: Contributions to inflammation-related diseases. Cell. Mol. Biol. Lett..

[16] Fiebich B.L., Batista C.R.A., Saliba S.W., Yousif N.M., de Oliveira A.C.P. (2018). Role of Microglia TLRs in Neurodegeneration. Front. Cell. Neurosci..

[17] Freeman L., Guo H., David C.N., Brickey W.J., Jha S., Ting J.P. (2017). NLR members NLRC4 and NLRP3 mediate sterile inflammasome activation in microglia and astrocytes. J. Exp. Med..

[18] Gradisnik L., Velnar T. (2023). Astrocytes in the central nervous system and their functions in health and disease: A review. World J. Clin. Cases.

[19] Nutma E., van Gent D., Amor S., Peferoen L.A.N. (2020). Astrocyte and Oligodendrocyte Cross-Talk in the Central Nervous System. Cells.

[20] Bouvier D.S., Fixemer S., Heurtaux T., Jeannelle F., Frauenknecht K.B.M., Mittelbronn M. (2022). The Multifaceted Neurotoxicity of Astrocytes in Ageing and Age-Related Neurodegenerative Diseases: A Translational Perspective. Front. Physiol..

[21] Manu D.R., Slevin M., Barcutean L., Forro T., Boghitoiu T., Balasa R. (2023). Astrocyte Involvement in Blood-Brain Barrier Function: A Critical Update Highlighting Novel, Complex, Neurovascular Interactions. Int. J. Mol Sci..

[22] Manich G., Recasens M., Valente T., Almolda B., González B., Castellano B. (2019). Role of the CD200-CD200R Axis During Homeostasis and Neuroinflammation. Neuroscience.

[23] Cook A., Hippensteel R., Shimizu S., Nicolai J., Fatatis A., Meucci O. (2010). Interactions between chemokines: Regulation of fractalkine/CX3CL1 homeostasis by SDF/CXCL12 in cortical neurons. J. Biol. Chem..

[24] Ledonne A., Mercuri N.B. (2019). On the Modulatory Roles of Neuregulins/ErbB Signaling on Synaptic Plasticity. Int. J. Mol. Sci..

[25] Müller L., Di Benedetto S., Müller V. (2025). From Homeostasis to Neuroinflammation: Insights into Cellular and Molecular Interactions and Network Dynamics. Cells.

[26] Bresnick A.R. (2018). S100 proteins as therapeutic targets. Biophys. Rev..

[27] Fritz G., Botelho H.M., Morozova-Roche L.A., Gomes C.M. (2010). Natural and amyloid self-assembly of S100 proteins: Structural basis of functional diversity. FEBS J..

[28] Zimmer D.B., Wright Sadosky P., Weber D.J. (2003). Molecular mechanisms of S100-target protein interactions. Microsc. Res. Tech..

[29] Singh P., Ali S.A. (2022). Multifunctional Role of S100 Protein Family in the Immune System: An Update. Cells.

[30] Heizmann C.W. (1999). Ca^2+^-binding S100 proteins in the central nervous system. Neurochem. Res..

[31] Donato R., Cannon B.R., Sorci G., Riuzzi F., Hsu K., Weber D.J., Geczy C.L. (2013). Functions of S100 proteins. Curr. Mol. Med..

[32] Chaturvedi N., Ahmad K., Yadav B.S., Lee E.J., Sonkar S.C., Marina N., Choi I. (2020). Understanding Calcium-Dependent Conformational Changes in S100A1 Protein: A Combination of Molecular Dynamics and Gene Expression Study in Skeletal Muscle. Cells.

[33] Völkers M., Rohde D., Goodman C., Most P. (2010). S100A1: A regulator of striated muscle sarcoplasmic reticulum Ca^2+^ handling, sarcomeric, and mitochondrial function. J. Biomed. Biotechnol..

[34] Mueller A., Schäfer B.W., Ferrari S., Weibel M., Makek M., Höchli M., Heizmann C.W. (2005). The calcium-binding protein S100A2 interacts with p53 and modulates its transcriptional activity. J. Biol. Chem..

[35] Pan S.C., Li C.Y., Kuo C.Y., Kuo Y.Z., Fang W.Y., Huang Y.H., Hsieh T.C., Kao H.Y., Kuo Y., Kang Y.R. (2018). The p53-S100A2 Positive Feedback Loop Negatively Regulates Epithelialization in Cutaneous Wound Healing. Sci. Rep..

[36] Guan W., Deng Q., Yu X.L., Yuan Y.S., Gao J., Li J.J., Zhou L., Xia P., Han G.Y., Han W. (2015). Blockade of S100A3 activity inhibits murine hair growth. Genet. Mol. Res..

[37] Li Z.H., Bresnick A.R. (2006). The S100A4 metastasis factor regulates cellular motility via a direct interaction with myosin-IIA. Cancer Res..

[38] Liu L., Qi L., Knifley T., Piecoro D.W., Rychahou P., Liu J., Mitov M.I., Martin J., Wang C., Wu J. (2019). S100A4 alters metabolism and promotes invasion of lung cancer cells by up-regulating mitochondrial complex I protein NDUFS2. J. Biol. Chem..

[39] Chan W.Y., Xia C.L., Dong D.C., Heizmann C.W., Yew D.T. (2003). Differential expression of S100 proteins in the developing human hippocampus and temporal cortex. Microsc. Res. Tech..

[40] Jurewicz E., Robaszkiewicz K., Moraczewska J., Filipek A. (2020). Binding of S100A6 to actin and the actin-tropomyosin complex. Sci. Rep..

[41] Bhatt T., Bhosale A., Bajantri B., Mathapathi M.S., Rizvi A., Scita G., Majumdar A., Jamora C. (2019). Sustained Secretion of the Antimicrobial Peptide S100A7 Is Dependent on the Downregulation of Caspase-8. Cell Rep..

[42] Emberley E.D., Alowami S., Snell L., Murphy L.C., Watson P.H. (2004). S100A7 (psoriasin) expression is associated with aggressive features and alteration of Jab1 in ductal carcinoma in situ of the breast. Breast Cancer Res..

[43] Wilkie T., Verma A.K., Zhao H., Charan M., Ahirwar D.K., Kant S., Pancholi V., Mishra S., Ganju R.K. (2022). Lipopolysaccharide from the commensal microbiota of the breast enhances cancer growth: Role of S100A7 and TLR4. Mol. Oncol..

[44] Ma L., Sun P., Zhang J.C., Zhang Q., Yao S.L. (2017). Proinflammatory effects of S100A8/A9 via TLR4 and RAGE signaling pathways in BV-2 microglial cells. Int. J. Mol. Med..

[45] Skronska-Wasek W., Durlanik S., Le H.Q., Schroeder V., Kitt K., Garnett J.P., Pflanz S. (2022). The antimicrobial peptide S100A8/A9 produced by airway epithelium functions as a potent and direct regulator of macrophage phenotype and function. Eur. Respir. J..

[46] Morel E., Gruenberg J. (2007). The p11/S100A10 light chain of annexin A2 is dispensable for annexin A2 association to endosomes and functions in endosomal transport. PLoS ONE.

[47] Miller V.A., Madureira P.A., Kamaludin A.A., Komar J., Sharma V., Sahni G., Thelwell C., Longstaff C., Waisman D.M. (2017). Mechanism of plasmin generation by S100A10. Thromb. Haemost..

[48] Davey G.E., Murmann P., Hoechli M., Tanaka T., Heizmann C.W. (2000). Calcium-dependent translocation of S100A11 requires tubulin filaments. Biochim. Biophys. Acta.

[49] Shin H., Lee J., Kim Y., Jang S., Lee Y., Kim S., Lee Y. (2017). Knockdown of BC200 RNA expression reduces cell migration and invasion by destabilizing mRNA for calcium-binding protein S100A11. RNA Biol..

[50] Zhang M.X., Gan W., Jing C.Y., Zheng S.S., Yi Y., Zhang J., Xu X., Lin J.J., Zhang B.H., Qiu S.J. (2019). S100A11 promotes cell proliferation via P38/MAPK signaling pathway in intrahepatic cholangiocarcinoma. Mol. Carcinog..

[51] Meijer B., Gearry R.B., Day A.S. (2012). The role of S100A12 as a systemic marker of inflammation. Int. J. Inflamm..

[52] Mikkelsen S.E., Novitskaya V., Kriajevska M., Berezin V., Bock E., Norrild B., Lukanidin E. (2001). S100A12 protein is a strong inducer of neurite outgrowth from primary hippocampal neurons. J. Neurochem..

[53] Landriscina M., Soldi R., Bagalá C., Micucci I., Bellum S., Tarantini F., Prudovsky I., Maciag T. (2001). S100A13 participates in the release of fibroblast growth factor 1 in response to heat shock in vitro. J. Biol. Chem..

[54] Mohan S.K., Yu C. (2011). The IL1alpha-S100A13 heterotetrameric complex structure: A component in the non-classical pathway for interleukin 1alpha secretion. J. Biol. Chem..

[55] Landriscina M., Schinzari G., Di Leonardo G., Quirino M., Cassano A., D’Argento E., Lauriola L., Scerrati M., Prudovsky I., Barone C. (2006). S100A13, a new marker of angiogenesis in human astrocytic gliomas. J. Neurooncol..

[56] Mandinova A., Soldi R., Graziani I., Bagala C., Bellum S., Landriscina M., Tarantini F., Prudovsky I., Maciag T. (2003). S100A13 mediates the copper-dependent stress-induced release of IL-1alpha from both human U937 and murine NIH 3T3 cells. J. Cell Sci..

[57] Zhao F.T., Jia Z.S., Yang Q., Song L., Jiang X.J. (2013). S100A14 promotes the growth and metastasis of hepatocellular carcinoma. Asian Pac. J. Cancer Prev..

[58] Jiang S., Zhu Y., Chen Z., Huang Z., Liu B., Xu Y., Li Z., Lin Z., Li M. (2021). S100A14 inhibits cell growth and epithelial-mesenchymal transition (EMT) in prostate cancer through FAT1-mediated Hippo signaling pathway. Hum. Cell.

[59] Wolf R., Lewerenz V., Büchau A.S., Walz M., Ruzicka T. (2007). Human S100A15 splice variants are differentially expressed in inflammatory skin diseases and regulated through Th1 cytokines and calcium. Exp. Dermatol..

[60] Liu Y., Zhang R., Xin J., Sun Y., Li J., Wei D., Zhao A.Z. (2011). Identification of S100A16 as a novel adipogenesis promoting factor in 3T3-L1 cells. Endocrinology.

[61] Xiang Y.Y., Liu J.H., Yi X., Luo J.Y., Yu Y., Yi G.L. (2025). S100 A16 promotes the progression of osteosarcoma by activating the PI3 K/AKT signaling pathway through ANXA2. Sci. Rep..

[62] Seguella L., Capuano R., Pesce M., Annunziata G., Pesce M., de Conno B., Sarnelli G., Aurino L., Esposito G. (2019). S100B Protein Stimulates Proliferation and Angiogenic Mediators Release through RAGE/pAkt/mTOR Pathway in Human Colon Adenocarcinoma Caco-2 Cells. Int. J. Mol. Sci..

[63] Lin J., Yang Q., Wilder P.T., Carrier F., Weber D.J. (2010). The calcium-binding protein S100B down-regulates p53 and apoptosis in malignant melanoma. J. Biol. Chem..

[64] Rezaei O., Pakdaman H., Gharehgozli K., Simani L., Vahedian-Azimi A., Asaadi S., Sahraei Z., Hajiesmaeili M. (2017). S100 B: A new concept in neurocritical care. Iran J. Neurol..

[65] Hong E.J., Jeung E.B. (2013). Biological significance of calbindin-D_9k_ within duodenal epithelium. Int. J. Mol. Sci..

[66] Cong Y., Cui Y., Wang S., Jiang L., Cao J., Zhu S., Birkin E., Lane J., Ruge F., Jiang W.G. (2020). Calcium-Binding Protein S100P Promotes Tumor Progression but Enhances Chemosensitivity in Breast Cancer. Front. Oncol..

[67] Barry S., Chelala C., Lines K., Sunamura M., Wang A., Marelli-Berg F.M., Brennan C., Lemoine N.R., Crnogorac-Jurcevic T. (2013). S100P is a metastasis-associated gene that facilitates transendothelial migration of pancreatic cancer cells. Clin. Exp. Metastasis.

[68] Gribenko A.V., Hopper J.E., Makhatadze G.I. (2001). Molecular characterization and tissue distribution of a novel member of the S100 family of EF-hand proteins. Biochemistry.

[69] Gonzalez L.L., Garrie K., Turner M.D. (2020). Role of S100 proteins in health and disease. Biochim. Biophys. Acta.

[70] Sreejit G., Flynn M.C., Patil M., Krishnamurthy P., Murphy A.J., Nagareddy P.R. (2020). S100 family proteins in inflammation and beyond. Adv. Clin. Chem..

[71] Abdi W., Romasco A., Alkurdi D., Santacruz E., Okinedo I., Zhang Y., Kannan S., Shakiba S., Richmond J.M. (2024). An overview of S100 proteins and their functions in skin homeostasis, interface dermatitis conditions and other skin pathologies. Exp. Dermatol..

[72] Zimmer D.B., Cornwall E.H., Reynolds P.D., Donald C.M. (1998). S100A1 regulates neurite organization, tubulin levels, and proliferation in PC12 cells. J. Biol. Chem..

[73] Wang H., Mao X., Ye L., Cheng H., Dai X. (2022). The Role of the S100 Protein Family in Glioma. J. Cancer.

[74] Hernández-Ortega K., Canul-Euan A.A., Solis-Paredes J.M., Borboa-Olivares H., Reyes-Muñoz E., Estrada-Gutierrez G., Camacho-Arroyo I. (2024). S100B actions on glial and neuronal cells in the developing brain: An overview. Front. Neurosci..

[75] Santamaria-Kisiel L., Rintala-Dempsey A.C., Shaw G.S. (2006). Calcium-dependent and -independent interactions of the S100 protein family. Biochem. J..

[76] Hermann A., Donato R., Weiger T.M., Chazin W.J. (2012). S100 calcium binding proteins and ion channels. Front. Pharmacol..

[77] Xia C., Braunstein Z., Toomey A.C., Zhong J., Rao X. (2018). S100 Proteins As an Important Regulator of Macrophage Inflammation. Front. Immunol..

[78] Permyakov S.E., Denesyuk A.I., Denessiouk K.A., Permyakova M.E., Kazakov A.S., Ismailov R.G., Rastrygina V.A., Sokolov A.S., Permyakov E.A. (2019). Monomeric state of S100P protein: Experimental and molecular dynamics study. Cell Calcium.

[79] Sivaraja V., Kumar T.K., Prudovsky I., Yu C. (2005). Three-dimensional solution structure of a unique S100 protein. Biochem. Biophys. Res. Commun..

[80] Gifford J.L., Walsh M.P., Vogel H.J. (2007). Structures and metal-ion-binding properties of the Ca^2+^-binding helix-loop-helix EF-hand motifs. Biochem. J..

[81] Yap K.L., Ames J.B., Swindells M.B., Ikura M. (1999). Diversity of conformational states and changes within the EF-hand protein superfamily. Proteins.

[82] Denessiouk K., Permyakov S., Denesyuk A., Permyakov E., Johnson M.S. (2014). Two structural motifs within canonical EF-hand calcium-binding domains identify five different classes of calcium buffers and sensors. PLoS ONE.

[83] Sattar Z., Lora A., Jundi B., Railwah C., Geraghty P. (2021). The S100 Protein Family as Players and Therapeutic Targets in Pulmonary Diseases. Pulm. Med..

[84] Moore B.W. (1965). A soluble protein characteristic of the nervous system. Biochem. Biophys. Res. Commun..

[85] Kiss B., Ecsédi P., Simon M., Nyitray L. (2019). Isolation and Characterization of S100 Protein-Protein Complexes. Calcium-Binding Proteins of the EF-Hand Superfamily: From Basics to Medical Applications.

[86] Zimmer D.B., Eubanks J.O., Ramakrishnan D., Criscitiello M.F. (2013). Evolution of the S100 family of calcium sensor proteins. Cell Calcium.

[87] Sedaghat F., Notopoulos A. (2008). S100 protein family and its application in clinical practice. Hippokratia.

[88] Liu M., Wang Y., Miettinen J.J., Kumari R., Majumder M.M., Tierney C., Bazou D., Parsons A., Suvela M., Lievonen J. (2021). S100 Calcium Binding Protein Family Members Associate With Poor Patient Outcome and Response to Proteasome Inhibition in Multiple Myeloma. Front. Cell Dev. Biol..

[89] Marenholz I., Volz A., Ziegler A., Davies A., Ragoussis I., Korge B.P., Mischke D. (1996). Genetic analysis of the epidermal differentiation complex (EDC) on human chromosome 1q21: Chromosomal orientation, new markers, and a 6-Mb YAC contig. Genomics.

[90] Kizawa K., Takahara H., Unno M., Heizmann C.W. (2011). S100 and S100 fused-type protein families in epidermal maturation with special focus on S100A3 in mammalian hair cuticles. Biochimie.

[91] Holthaus K.B., Sachslehner A.P., Steinbinder J., Eckhart L. (2024). Epidermal Differentiation Genes of the Common Wall Lizard Encode Proteins with Extremely Biased Amino Acid Contents. Genes.

[92] Shang X., Cheng H., Zhou R. (2008). Chromosomal mapping, differential origin and evolution of the S100 gene family. Genet. Sel. Evol..

[93] Cross S.S., Hamdy F.C., Deloulme J.C., Rehman I. (2005). Expression of S100 proteins in normal human tissues and common cancers using tissue microarrays: S100A6, S100A8, S100A9 and S100A11 are all overexpressed in common cancers. Histopathology.

[94] Leśniak W. (2011). Epigenetic regulation of S100 protein expression. Clin. Epigenetics.

[95] Lindsey J.C., Lusher M.E., Anderton J.A., Gilbertson R.J., Ellison D.W., Clifford S.C. (2007). Epigenetic deregulation of multiple S100 gene family members by differential hypomethylation and hypermethylation events in medulloblastoma. Br. J. Cancer.

[96] Mossel D.M., Moganti K., Riabov V., Weiss C., Kopf S., Cordero J., Dobreva G., Rots M.G., Klüter H., Harmsen M.C. (2020). Epigenetic Regulation of S100A9 and S100A12 Expression in Monocyte-Macrophage System in Hyperglycemic Conditions. Front. Immunol..

[97] Hofmann M.A., Drury S., Fu C., Qu W., Taguchi A., Lu Y., Avila C., Kambham N., Bierhaus A., Nawroth P. (1999). RAGE mediates a novel proinflammatory axis: A central cell surface receptor for S100/calgranulin polypeptides. Cell.

[98] Leclerc E., Vetter S.W. (2015). The role of S100 proteins and their receptor RAGE in pancreatic cancer. Biochim. Biophys. Acta..

[99] Ray R., Juranek J.K., Rai V. (2016). RAGE axis in neuroinflammation, neurodegeneration and its emerging role in the pathogenesis of amyotrophic lateral sclerosis. Neurosci. Biobehav. Rev..

[100] Leclerc E., Fritz G., Vetter S.W., Heizmann C.W. (2009). Binding of S100 proteins to RAGE: An update. Biochim. Biophys. Acta.

[101] Penumutchu S.R., Chou R.H., Yu C. (2014). Structural insights into calcium-bound S100P and the V domain of the RAGE complex. PLoS ONE.

[102] Lin H., Andersen G.R., Yatime L. (2016). Crystal structure of human S100A8 in complex with zinc and calcium. BMC Struct. Biol..

[103] Koch M., Chitayat S., Dattilo B.M., Schiefner A., Diez J., Chazin W.J., Fritz G. (2010). Structural basis for ligand recognition and activation of RAGE. Structure.

[104] Hudson B.I., Lippman M.E. (2018). Targeting RAGE Signaling in Inflammatory Disease. Annu. Rev. Med..

[105] Sparvero L.J., Asafu-Adjei D., Kang R., Tang D., Amin N., Im J., Rutledge R., Lin B., Amoscato A.A., Zeh H.J. (2009). RAGE (Receptor for Advanced Glycation Endproducts), RAGE ligands, and their role in cancer and inflammation. J. Transl. Med..

[106] Arivazhagan L., Popp C.J., Ruiz H.H., Wilson R.A., Manigrasso M.B., Shekhtman A., Ramasamy R., Sevick M.A., Schmidt A.M. (2024). The RAGE/DIAPH1 axis: Mediator of obesity and proposed biomarker of human cardiometabolic disease. Cardiovasc. Res..

[107] Ramasamy R., Shekhtman A., Schmidt A.M. (2022). The RAGE/DIAPH1 Signaling Axis & Implications for the Pathogenesis of Diabetic Complications. Int. J. Mol. Sci..

[108] Tóbon-Velasco J.C., Cuevas E., Torres-Ramos M.A. (2014). Receptor for AGEs (RAGE) as mediator of NF-kB pathway activation in neuroinflammation and oxidative stress. CNS Neurol. Disord. Drug Targets.

[109] Hinz M., Scheidereit C. (2014). The IκB kinase complex in NF-κB regulation and beyond. EMBO Rep..

[110] Wang X., Peng H., Huang Y., Kong W., Cui Q., Du J., Jin H. (2020). Post-translational Modifications of IκBα: The State of the Art. Front. Cell Dev. Biol..

[111] Kroll M., Margottin F., Kohl A., Renard P., Durand H., Concordet J.P., Bachelerie F., Arenzana-Seisdedos F., Benarous R. (1999). Inducible degradation of IkappaBalpha by the proteasome requires interaction with the F-box protein h-betaTrCP. J. Biol. Chem..

[112] Florio T.J., Lokareddy R.K., Yeggoni D.P., Sankhala R.S., Ott C.A., Gillilan R.E., Cingolani G. (2022). Differential recognition of canonical NF-κB dimers by Importin α3. Nat. Commun..

[113] Wang V.Y., Huang W., Asagiri M., Spann N., Hoffmann A., Glass C., Ghosh G. (2012). The transcriptional specificity of NF-κB dimers is coded within the κB DNA response elements. Cell Rep..

[114] Mulero M.C., Wang V.Y., Huxford T., Ghosh G. (2019). Genome reading by the NF-κB transcription factors. Nucleic Acids Res..

[115] Liu T., Zhang L., Joo D., Sun S.C. (2017). NF-κB signaling in inflammation. Signal Transduct. Target. Ther..

[116] Pan Y., Zhang X., Wang Y., Cai L., Ren L., Tang L., Wang J., Zhao Y., Wang Y., Liu Q. (2013). Targeting JNK by a new curcumin analog to inhibit NF-kB-mediated expression of cell adhesion molecules attenuates renal macrophage infiltration and injury in diabetic mice. PLoS ONE.

[117] Parrondo R., de las Pozas A., Reiner T., Rai P., Perez-Stable C. (2010). NF-κB activation enhances cell death by antimitotic drugs in human prostate cancer cells. Mol. Cancer.

[118] Lingappan K. (2018). NF-κB in Oxidative Stress. Curr. Opin. Toxicol..

[119] Zglejc-Waszak K., Pomianowski A., Wojtkiewicz J., Banach M., Juranek J.K. (2023). New insights into RAGE/Diaph1 interaction as a modulator of actin cytoskeleton dynamics in peripheral nervous system in long-term hyperglycaemia. Eur. J. Neurosci..

[120] Zhu P., Ren M., Yang C., Hu Y.X., Ran J.M., Yan L. (2012). Involvement of RAGE, MAPK and NF-κB pathways in AGEs-induced MMP-9 activation in HaCaT keratinocytes. Exp. Dermatol..

[121] Bao J.M., He M.Y., Liu Y.W., Lu Y.J., Hong Y.Q., Luo H.H., Ren Z.L., Zhao S.C., Jiang Y. (2015). AGE/RAGE/Akt pathway contributes to prostate cancer cell proliferation by promoting Rb phosphorylation and degradation. Am. J. Cancer Res..

[122] Serban A.I., Stanca L., Geicu O.I., Dinischiotu A. (2015). AGEs-Induced IL-6 Synthesis Precedes RAGE Up-Regulation in HEK 293 Cells: An Alternative Inflammatory Mechanism?. Int. J. Mol. Sci..

[123] Lim S.Y., Raftery M.J., Goyette J., Hsu K., Geczy C.L. (2009). Oxidative modifications of S100 proteins: Functional regulation by redox. J. Leukoc. Biol..

[124] Lee J.M., Hammarén H.M., Savitski M.M., Baek S.H. (2023). Control of protein stability by post-translational modifications. Nat. Commun..

[125] Seitz A., Busch M., Kroemer J., Schneider A., Simon S., Jungmann A., Katus H.A., Most P., Ritterhoff J. (2024). S100A1’s single cysteine is an indispensable redox switch for the protection against diastolic calcium waves in cardiomyocytes. Am. J. Physiol. Heart Circ. Physiol..

[126] Zaręba-Kozioł M., Burdukiewicz M., Wysłouch-Cieszyńska A. (2022). Intracellular Protein S-Nitrosylation—A Cells Response to Extracellular S100B and RAGE Receptor. Biomolecules.

[127] Malik P., Kumar Mukherjee T. (2022). Immunological methods for the determination of AGE-RAGE axis generated glutathionylated and carbonylated proteins as oxidative stress markers. Methods.

[128] Piras S., Furfaro A.L., Domenicotti C., Traverso N., Marinari U.M., Pronzato M.A., Nitti M. (2016). RAGE Expression and ROS Generation in Neurons: Differentiation versus Damage. Oxidative Med. Cell. Longev..

[129] Yamaguchi F., Umeda Y., Shimamoto S., Tsuchiya M., Tokumitsu H., Tokuda M., Kobayashi R. (2012). S100 proteins modulate protein phosphatase 5 function: A link between CA^2+^ signal transduction and protein dephosphorylation. J. Biol. Chem..

[130] Downs C.A., Kreiner L.H., Johnson N.M., Brown L.A., Helms M.N. (2015). Receptor for advanced glycation end-products regulates lung fluid balance via protein kinase C-gp91^phox^ signaling to epithelial sodium channels. Am. J. Respir. Cell Mol. Biol..

[131] Coste K., Bruet S., Chollat-Namy C., Filhol O., Cochet C., Gallot D., Marceau G., Blanchon L., Sapin V., Belville C. (2023). Characterization of RAGE and CK2 Expressions in Human Fetal Membranes. Int. J. Mol. Sci..

[132] Dong W., Yang X., Li X., Wei S., An C., Zhang J., Shi X., Dong S. (2024). Investigation of N-Glycan Functions in Receptor for Advanced Glycation End Products V Domain through Chemical Glycoprotein Synthesis. J. Am. Chem. Soc..

[133] Degani G., Barbiroli A., Magnelli P., Digiovanni S., Altomare A., Aldini G., Popolo L. (2019). Insights into the effects of N-glycosylation on the characteristics of the VC1 domain of the human receptor for advanced glycation end products (RAGE) secreted by Pichia pastoris. Glycoconj. J..

[134] Bertheloot D., Latz E. (2017). HMGB1, IL-1α, IL-33 and S100 proteins: Dual-function alarmins. Cell. Mol. Immunol..

[135] Barger S.W., Van Eldik L.J. (1992). S100 beta stimulates calcium fluxes in glial and neuronal cells. J. Biol. Chem..

[136] Lancaster T., Tabrizi M.E.A., Repici M., Gupta J., Gross S.R. (2023). An Extracellular/Membrane-Bound S100P Pool Regulates Motility and Invasion of Human Extravillous Trophoblast Lines and Primary Cells. Biomolecules.

[137] Hu J., Van Eldik L.J. (1996). S100 beta induces apoptotic cell death in cultured astrocytes via a nitric oxide-dependent pathway. Biochim. Biophys. Acta.

[138] Ghavami S., Eshragi M., Ande S.R., Chazin W.J., Klonisch T., Halayko A.J., McNeill K.D., Hashemi M., Kerkhoff C., Los M. (2010). S100A8/A9 induces autophagy and apoptosis via ROS-mediated cross-talk between mitochondria and lysosomes that involves BNIP3. Cell Res..

[139] Taneja S., Vetter S.W., Leclerc E. (2021). Hypoxia and the Receptor for Advanced Glycation End Products (RAGE) Signaling in Cancer. Int. J. Mol. Sci..

[140] Fernandez-Fernandez M.R., Rutherford T.J., Fersht A.R. (2008). Members of the S100 family bind p53 in two distinct ways. Protein Sci..

[141] Ishijima T., Nakajima K. (2021). Inflammatory cytokines TNFα, IL-1β, and IL-6 are induced in endotoxin- stimulated microglia through different signaling cascades. Sci. Prog..

[142] Nam A.R., Kim D.H., Kim M.J., Lee J.S., Yang S.J., Kim I.S. (2016). S100A8 Induces Secretion of MCP-1, IL-6, and IL-8 via TLR4 in Jurkat T Cells. Biomed. Sci. Lett..

[143] Noack M., Miossec P. (2023). Heterogeneous effects of S100 proteins during cell interactions between immune cells and stromal cells from synovium or skin. Clin. Exp. Immunol..

[144] Kazakov A.S., Rastrygina V.A., Vologzhannikova A.A., Zemskova M.Y., Bobrova L.A., Deryusheva E.I., Permyakova M.E., Sokolov A.S., Litus E.A., Shevelyova M.P. (2024). Recognition of granulocyte-macrophage colony-stimulating factor by specific S100 proteins. Cell Calcium.

[145] Kushi H., Saito T., Makino K., Hayashi N. (2003). L-8 is a key mediator of neuroinflammation in severe traumatic brain injuries. Brain Edema XII.

[146] Chen J., Liu X., Zhong Y. (2020). Interleukin-17A: The Key Cytokine in Neurodegenerative Diseases. Front. Aging Neurosci..

[147] Croxford A.L., Spath S., Becher B. (2015). GM-CSF in Neuroinflammation: Licensing Myeloid Cells for Tissue Damage. Trends Immunol..

[148] Gross S.R., Sin C.G., Barraclough R., Rudland P.S. (2014). Joining S100 proteins and migration: For better or for worse, in sickness and in health. Cell. Mol. Life Sci..

[149] Bai X., Xu P.C., Chen T., Zhang H.M., Wu S.J., Yang X., Gao S., Jia J.Y., Jiang J.Q., Yan T.K. (2022). The potential pathogenic roles of S100A8/A9 and S100A12 in patients with MPO-ANCA-positive vasculitis. BMC Immunol..

[150] Yoshinouchi T., Ohtsuki Y., Ueda R., Sato S., Ueda N. (1999). Myofibroblasts and S-100 protein positive cells in idiopathic pulmonary fibrosis and rheumatoid arthritis-associated interstitial pneumonia. Eur. Respir. J..

[151] Lallyett C., Yeung C.C., Nielson R.H., Zeef L.A.H., Chapman-Jones D., Kjaer M., Kadler K.E. (2018). Changes in S100 Proteins Identified in Healthy Skin following Electrical Stimulation: Relevance for Wound Healing. Adv. Ski. Wound Care.

[152] Cristóvão J.S., Gomes C.M. (2019). S100 Proteins in Alzheimer’s Disease. Front. Neurosci..

[153] Angelopoulou E., Paudel Y.N., Piperi C. (2021). Emerging role of S100B protein implication in Parkinson’s disease pathogenesis. Cell. Mol. Life Sci..

[154] Holzinger D., Foell D., Kessel C. (2018). The role of S100 proteins in the pathogenesis and monitoring of autoinflammatory diseases. Mol. Cell. Pediatr..

[155] Bresnick A.R., Weber D.J., Zimmer D.B. (2015). S100 proteins in cancer. Nat. Rev. Cancer.

[156] Ackermann G.E., Marenholz I., Wolfer D.P., Chan W.Y., Schäfer B., Erne P., Heizmann C.W. (2006). S100A1-deficient male mice exhibit increased exploratory activity and reduced anxiety-related responses. Biochim. Biophys. Acta.

[157] Filipek A., Leśniak W. (2020). S100A6 and Its Brain Ligands in Neurodegenerative Disorders. Int. J. Mol. Sci..

[158] Tian Q., Li Z., Yan Z., Jiang S., Zhao X., Wang L., Li M. (2024). Inflammatory role of S100A8/A9 in the central nervous system non-neoplastic diseases. Brain Res. Bull..

[159] Shepherd C.E., Goyette J., Utter V., Rahimi F., Yang Z., Geczy C.L., Halliday G.M. (2006). Inflammatory S100A9 and S100A12 proteins in Alzheimer’s disease. Neurobiol. Aging.

[160] Steiner J., Bogerts B., Schroeter M.L., Bernstein H.G. (2011). S100B protein in neurodegenerative disorders. Clin. Chem. Lab. Med..

[161] Brozzi F., Arcuri C., Giambanco I., Donato R. (2009). S100B Protein Regulates Astrocyte Shape and Migration via Interaction with Src Kinase: IMPLICATIONS FOR ASTROCYTE DEVELOPMENT, ACTIVATION, AND TUMOR GROWTH. J. Biol. Chem..

[162] Hoyaux D., Boom A., Van den Bosch L., Belot N., Martin J.J., Heizmann C.W., Kiss R., Pochet R. (2002). S100A6 overexpression within astrocytes associated with impaired axons from both ALS mouse model and human patients. J. Neuropathol. Exp. Neurol..

[163] Wu M., Xu L., Wang Y., Zhou N., Zhen F., Zhang Y., Qu X., Fan H., Liu S., Chen Y. (2018). S100A8/A9 induces microglia activation and promotes the apoptosis of oligodendrocyte precursor cells by activating the NF-κB signaling pathway. Brain Res. Bull..

[164] Dong N., Wang Y. (2019). MiR-30a Regulates S100A12-induced Retinal Microglial Activation and Inflammation by Targeting NLRP3. Curr. Eye Res..

[165] Lisachev P.D., Shtark M.B., Sokolova O.O., Pustylnyak V.O., Salakhutdinova M.Y., Epstein O.I. (2010). A Comparison of the Dynamics of S100B, S100A1, and S100A6 mRNA Expression in Hippocampal CA1 Area of Rats during Long-Term Potentiation and after Low-Frequency Stimulation. Cardiovasc. Psychiatry Neurol..

[166] Rickmann M., Wolff J.R. (1995). S100 protein expression in subpopulations of neurons of rat brain. Neuroscience.

[167] Leśniak W., Filipek A. (2024). S100 Proteins—Intracellular and Extracellular Function in Norm and Pathology. Biomolecules.

[168] Donato R., Sorci G., Riuzzi F., Arcuri C., Bianchi R., Brozzi F., Tubaro C., Giambanco I. (2009). S100B’s double life: Intracellular regulator and extracellular signal. Biochim. Biophys. Acta.

[169] Michetti F., Clementi M.E., Di Liddo R., Valeriani F., Ria F., Rende M., Di Sante G., Romano Spica V. (2023). The S100B Protein: A Multifaceted Pathogenic Factor More Than a Biomarker. Int. J. Mol. Sci..

[170] Wright N.T., Cannon B.R., Zimmer D.B., Weber D.J. (2009). S100A1: Structure, Function, and Therapeutic Potential. Curr. Chem. Biol..

[171] Donato R., Sorci G., Giambanco I. (2017). S100A6 protein: Functional roles. Cell. Mol. Life Sci..

[172] Viemann D. (2020). S100-Alarmins Are Essential Pilots of Postnatal Innate Immune Adaptation. Front. Immunol..

[173] Kleissner M., Sramko M., Kohoutek J., Kautzner J., Kettner J. (2021). Serum S100 Protein Is a Reliable Predictor of Brain Injury After Out-of-Hospital Cardiac Arrest: A Cohort Study. Front. Cardiovasc. Med..

[174] Singh A.K., Asif S., Pandey D.K., Chaudhary A., Kapoor V., Verma P.K. (2024). Biomarkers in Acute Traumatic Brain Injury: A Systematic Review and Meta-Analysis. Cureus.

[175] Lopes A.N., Regner A., Simon D. (2024). The Role of S100b Protein Biomarker in Brain Death: A Literature Review. Cureus.

[176] Camponeschi C., De Carluccio M., Amadio S., Clementi M.E., Sampaolese B., Volonté C., Tredicine M., Romano Spica V., Di Liddo R., Ria F. (2021). S100B Protein as a Therapeutic Target in Multiple Sclerosis: The S100B Inhibitor Arundic Acid Protects from Chronic Experimental Autoimmune Encephalomyelitis. Int. J. Mol. Sci..

[177] Migheli A., Cordera S., Bendotti C., Atzori C., Piva R., Schiffer D. (1999). S-100β protein is upregulated in astrocytes and motor neurons in the spinal cord of patients with amyotrophic lateral sclerosis. Neurosci. Lett..

[178] Hagmeyer S., Romão M.A., Cristóvão J.S., Vilella A., Zoli M., Gomes C.M., Grabrucker A.M. (2019). Distribution and Relative Abundance of S100 Proteins in the Brain of the APP23 Alzheimer’s Disease Model Mice. Front. Neurosci..

[179] Schuermans S., Kestens C., Marques P.E. (2024). Systemic mechanisms of necrotic cell debris clearance. Cell Death Dis..

[180] Heizmann C.W. (2019). S100 proteins: Diagnostic and prognostic biomarkers in laboratory medicine. Biochim. Biophys. Acta.

[181] Mori T., Koyama N., Arendash G.W., Horikoshi-Sakuraba Y., Tan J., Town T. (2010). Overexpression of human S100B exacerbates cerebral amyloidosis and gliosis in the Tg2576 mouse model of Alzheimer’s disease. GLIA.

[182] Mrak R.E., Griffinbc W.S. (2001). The role of activated astrocytes and of the neurotrophic cytokine S100B in the pathogenesis of Alzheimer’s disease. Neurobiol. Aging.

[183] Origlia N., Arancio O., Domenici L., Yan S.S. (2009). MAPK, beta-amyloid and synaptic dysfunction: The role of RAGE. Expert Rev. Neurother..

[184] Hampel H., Hardy J., Blennow K., Chen C., Perry G., Kim S.H., Villemagne V.L., Aisen P., Vendruscolo M., Iwatsubo T. (2021). The Amyloid-β Pathway in Alzheimer’s Disease. Mol. Psychiatry.

[185] Esposito G., Scuderi C., Lu J., Savani C., De Filippis D., Iuvone T., Steardo L., Sheen V., Steardo L. (2008). S100B induces tau protein hyperphosphorylation via Dickopff-1 up-regulation and disrupts the Wnt pathway in human neural stem cells. J. Cell. Mol. Med..

[186] Dugger B.N., Whiteside C.M., Maarouf C.L., Walker D.G., Beach T.G., Sue L.I., Garcia A., Dunckley T., Meechoovet B., Reiman E.M. (2016). The Presence of Select Tau Species in Human Peripheral Tissues and Their Relation to Alzheimer’s Disease. J. Alzheimers Dis..

[187] Litus E.A., Shevelyova M.P., Vologzhannikova A.A., Deryusheva E.I., Machulin A.V., Nemashkalova E.L., Permyakova M.E., Sokolov A.S., Alikova V.D., Uversky V.N. (2025). Binding of Pro-Inflammatory Proteins S100A8 or S100A9 to Amyloid-β Peptide Suppresses Its Fibrillation. Biomolecules.

[188] Lodeiro M., Puerta E., Ismail M.A., Rodriguez-Rodriguez P., Rönnbäck A., Codita A., Parrado-Fernandez C., Maioli S., Gil-Bea F., Merino-Serrais P. (2017). Aggregation of the Inflammatory S100A8 Precedes Aβ Plaque Formation in Transgenic APP Mice: Positive Feedback for S100A8 and Aβ Productions. J. Gerontol. Ser. A Biomed. Sci. Med. Sci..

[189] Wang C., Klechikov A.G., Gharibyan A.L., Wärmländer S.K., Jarvet J., Zhao L., Jia X., Narayana V.K., Shankar S.K., Olofsson A. (2014). The role of pro-inflammatory S100A9 in Alzheimer’s disease amyloid-neuroinflammatory cascade. Acta Neuropathol..

[190] Sathe K., Maetzler W., Lang J.D., Mounsey R.B., Fleckenstein C., Martin H.L., Schulte C., Mustafa S., Synofzik M., Vukovic Z. (2012). S100B is increased in Parkinson’s disease and ablation protects against MPTP-induced toxicity through the RAGE and TNF-α pathway. Brain.

[191] Reeve A.K., Ludtmann M.H., Angelova P.R., Simcox E.M., Horrocks M.H., Klenerman D., Gandhi S., Turnbull D.M., Abramov A.Y. (2015). Aggregated α-synuclein and complex I deficiency: Exploration of their relationship in differentiated neurons. Cell Death Dis..

[192] Puspita L., Chung S.Y., Shim J.W. (2017). Oxidative stress and cellular pathologies in Parkinson’s disease. Mol. Brain..

[193] Radi R. (2013). Peroxynitrite, a stealthy biological oxidant. J. Biol. Chem..

[194] Chinta S.J., Andersen J.K. (2008). Redox imbalance in Parkinson’s disease. Biochim. Biophys. Acta.

[195] Fardell C., Zettergren A., Ran C., Carmine Belin A., Ekman A., Sydow O., Bäckman L., Holmberg B., Dizdar N., Söderkvist P. (2018). S100B polymorphisms are associated with age of onset of Parkinson’s disease. BMC Med. Genet..

[196] Zervides K.A., Jern A., Nystedt J., Gullstrand B., Nilsson P.C., Sundgren P.C., Bengtsson A.A., Jönsen A. (2022). Serum S100A8/A9 concentrations are associated with neuropsychiatric involvement in systemic lupus erythematosus: A cross-sectional study. BMC Rheumatol..

[197] Wang S., Song R., Wang Z., Jing Z., Wang S., Ma J. (2018). S100A8/A9 in Inflammation. Front. Immunol..

[198] De Carluccio M., Di Sante G., Clementi M.E., Ruggirello M., Stabile A.M., Pistilli A., Marini S., Romano Spica V., Rende M., Ria F. (2025). Effect on Different Glial Cell Types of S100B Modulation in Multiple Sclerosis Experimental Models. Int. J. Mol. Sci..

[199] Schmidt S., Linington C., Zipp F., Sotgiu S., de Waal Malefyt R., Wekerle H., Hohlfeld R. (1997). Multiple sclerosis: Comparison of the human T-cell response to S100 beta and myelin basic protein reveals parallels to rat experimental autoimmune panencephalitis. Brain.

[200] Lovett-Racke A.E., Yang Y., Racke M.K. (2011). Th1 versus Th17: Are T cell cytokines relevant in multiple sclerosis?. Biochim. Biophys. Acta.

[201] Süssmuth S.D., Tumani H., Ecker D., Ludolph A.C. (2003). Amyotrophic lateral sclerosis: Disease stage related changes of tau protein and S100 beta in cerebrospinal fluid and creatine kinase in serum. Neurosci. Lett..

[202] Juranek J.K., Daffu G.K., Wojtkiewicz J., Lacomis D., Kofler J., Schmidt A.M. (2015). Receptor for Advanced Glycation End Products and its Inflammatory Ligands are Upregulated in Amyotrophic Lateral Sclerosis. Front. Cell. Neurosci..

[203] Kamo H., Haebara H., Akiguchi I., Kameyama M., Kimura H., McGeer P.L. (1987). A distinctive distribution of reactive astroglia in the precentral cortex in amyotrophic lateral sclerosis. Acta Neuropathol..

[204] Dıaz-Amarilla P., Olivera-Bravo S., Trias E., Cragnolini A., MartınezPalma L., Cassina P., Beckman J., Barbeito L. (2011). Phenotypically aberrant astrocytes that promote motoneuron damage in a model of inherited amyotrophic lateral sclerosis. Proc. Natl. Acad. Sci. USA.

[205] Serrano A., Donno C., Giannetti S., Peric M., Andjus P., D’Ambrosi N., Michetti F. (2017). The astrocytic S100B protein with its receptor RAGE is aberrantly expressed in SOD1^G93A^ models, and its inhibition decreases the expression of proinflammatory genes. Mediat. Inflamm..

[206] Hu J., Ferreira A., Van Eldik L.J. (1997). S100β induces neuronal cell death through nitric oxide release from astrocytes. J. Neurochem..

[207] Koh S.X., Lee J.K. (2014). S100B as a marker for brain damage and blood-brain barrier disruption following exercise. Sports Med..

[208] Steinruecke M., Lonergan R.M., Selvaraj B.T., Chandran S., Diaz-Castro B., Stavrou M. (2023). Blood-CNS barrier dysfunction in amyotrophic lateral sclerosis: Proposed mechanisms and clinical implications. J. Cereb. Blood Flow Metab..

[209] Thelin E.P., Nelson D.W., Bellander B.M. (2017). A review of the clinical utility of serum S100B protein levels in the assessment of traumatic brain injury. Acta Neurochir..

[210] Janigro D., Mondello S., Posti J.P., Unden J. (2022). GFAP and S100B: What You Always Wanted to Know and Never Dared to Ask. Front. Neurol..

[211] Oris C., Kahouadji S., Durif J., Bouvier D., Sapin V. (2023). S100B, Actor and Biomarker of Mild Traumatic Brain Injury. Int. J. Mol. Sci..

[212] Dmytriyeva O., Pankratova S., Owczarek S., Sonn K., Soroka V., Ridley C.M., Marsolais A., Lopez-Hoyos M., Ambartsumian N., Lukanidin E. (2012). The metastasis-promoting S100A4 protein confers neuroprotection in brain injury. Nat. Commun..

[213] Fang B., Liang M., Yang G., Ye Y., Xu H., He X., Huang J.H. (2014). Expression of S100A6 in rat hippocampus after traumatic brain injury due to lateral head acceleration. Int. J. Mol. Sci..

[214] He G.Y., Zhao C.H., Wu D.G., Cheng H., Sun L.A., Zhang D.L., Yang X.J., Fan X.R., Di G.F., Jiang X.C. (2021). S100A8 Promotes Inflammation via Toll-Like Receptor 4 After Experimental Traumatic Brain Injury. Front. Neurosci..

